# The next-generation sequencing reveals the complete mitochondrial genome of *Alosa sapidissima* (Perciformes: Clupeidae) with phylogenetic consideration

**DOI:** 10.1080/23802359.2017.1331322

**Published:** 2017-05-25

**Authors:** Jing Wang, Zeshu Yu, Xiang Wang, Shaosheng Yang, Dongguo Zhang, Yong Zhang

**Affiliations:** a State Key Laboratory of Biocontrol, Institute of Aquatic Economic Animals, The Guangdong Province Key Laboratory for Aquatic Economic Animals, Sun Yat-Sen University, Guangzhou, China;; b Shenzhen South Ocean Technology Co. Ltd, Shenzhen, China

**Keywords:** *Alosa sapidissima*, mitochondrial genome, phylogenetic analysis

## Abstract

The complete mitochondrial genome of the *Alosa sapidissima* is presented in this study. The mitochondrial genome is 16,741 bp long and consists of 13 protein-coding genes, 2 rRNA genes, 22 tRNA genes, and a control region. The gene order and composition of Epinephelus awoara mitochondrial genome was similar to that of most other vertebrates. The nucleotide compositions of the light strand in descending order is 28.79% of G, 28.39% of T, 24.94% of A, and 17.88% of C. With the exception of the NADH dehydrogenase subunit 6 (ND6) and 8 tRNA genes, all other mitochondrial genes are encoded on the heavy strand. The phylogenetic analysis by maximum-likelihood (ML) method showed that the *A. sapidissima* has the closer relationship to between the *A. alosa* and *A. pseudoharengus* in the phylogenetic relationship.

The American shad, *Alosa sapidissima*, is a eurythermic migratory fish which can survive in fresh and brackish water. It is distributed in the Mississippi River and the eastern coastal waters of North America (Rasmussen et al. 2011). *Alosa sapidissima* is rich in protein and unsaturated fatty acids, also contains calcium, iron, phosphorus, and magnesium; its docosahexenoic acid (DHA) content is much higher than the general freshwater fish. Thus, it poses a high nutritional value and health care value (Guo et al. 2010). However, the genetic information of *A. sapidissima* is very little. The next-generation sequencing (NGS) technologies, such as Illumine, allow considerable numbers of sequence data to be rapidly and efficiently characterized, which make it particularly feasible for mitogenomes (Gilbert et al. 2007). Moreover, Illumine has been successfully used to assemble the mitogenomes of fish species (Cui et al. 2009). Therefore, we determined to sequence the complete mitochondrial genome of *A. sapidissima* using the next-generation sequencing (NGS) techniques strategy in order to find new DNA markers for the studies on population genetics of *A. sapidissima*. The specimen was obtained from the Daya Bay Aquaculture Center, Guangdong, China. Then the specimen was preserved in 95% ethanol. The total genomic DNA was extracted from the fin of the fresh fish using the salting-out procedure (Howe et al. 1997).

The complete mitochondrial genome of *A. sapidissima* (Genbank accession number KY769128) is 16,741 bp in length, consisting of 13 protein-coding genes, 2 ribosomal RNA genes (12S rRNA and 16S rRNA), 22 transfer RNA genes (tRNA), and 1 control region, which is the same as the typical vertebrates (Wang et al. 2008). Most of the genes are encoded on the heavy strand, with only the NADH dehydrogenase subunit 6 (ND6) and eight tRNA genes [Gln, Ala, Asn, Cys, Try, Glu, Pro, Ser (TGA)] encoded on the light strand. Overall nucleotide compositions of the light strand are 24.94% of A, 17.88% of C, 28.39% of T, and 28.79% of G. However, the most representative base is T and the bias against C was observed, which was similar to the base compositions of mitochondrial genome of other teleosts.

All the protein-coding genes begin with an ATG start codon except for COX1 started with GTG. Three types of stop codons revealed are TAA (COX1, ATP8, ATP6, COXIII, ND4L, ND5), TAG (ND1, ND2, ND3, ND6), and T (COXII, ND4, CYTb). The 12S and 16S rRNA genes are located between the tRNA-Phe (GAA) and tRNA-Leu (TAA) genes, and are separated by the tRNA-Val gene with the same situation found in other vertebrates. Most genes are either abutted or overlapped. The 22 tRNA genes vary from 67 to 73 bp in length. All these could be folded into the typical cloverleaf secondary structure although numerous non-complementary and T–G base pairs exist in the stem regions. The control region was 1082 bp in length, located between tRNA-Pro (TGG) and tRNA-Phe (GAA) gene. The nucleotide composition of control region was 33.64% of A, 20.43% of C, 14.88% of G, 31.05% of T.

The phylogenetic position of *A. sapidissima* was reconstructed with the complete mtDNA sequences from 28 species of Perciformes by using the maximum-likelihood (ML) methods (Kumar et al. 2004). As shown in Figure 1, the *A. sapidissima* has the closer relationship to *A. alosa* and *A. pseudoharengus*. Furthermore, the *A. alosa* and *A. alabamae* clustered into a monophyletic group suggested the closer relationship between them.

**Figure 1.**
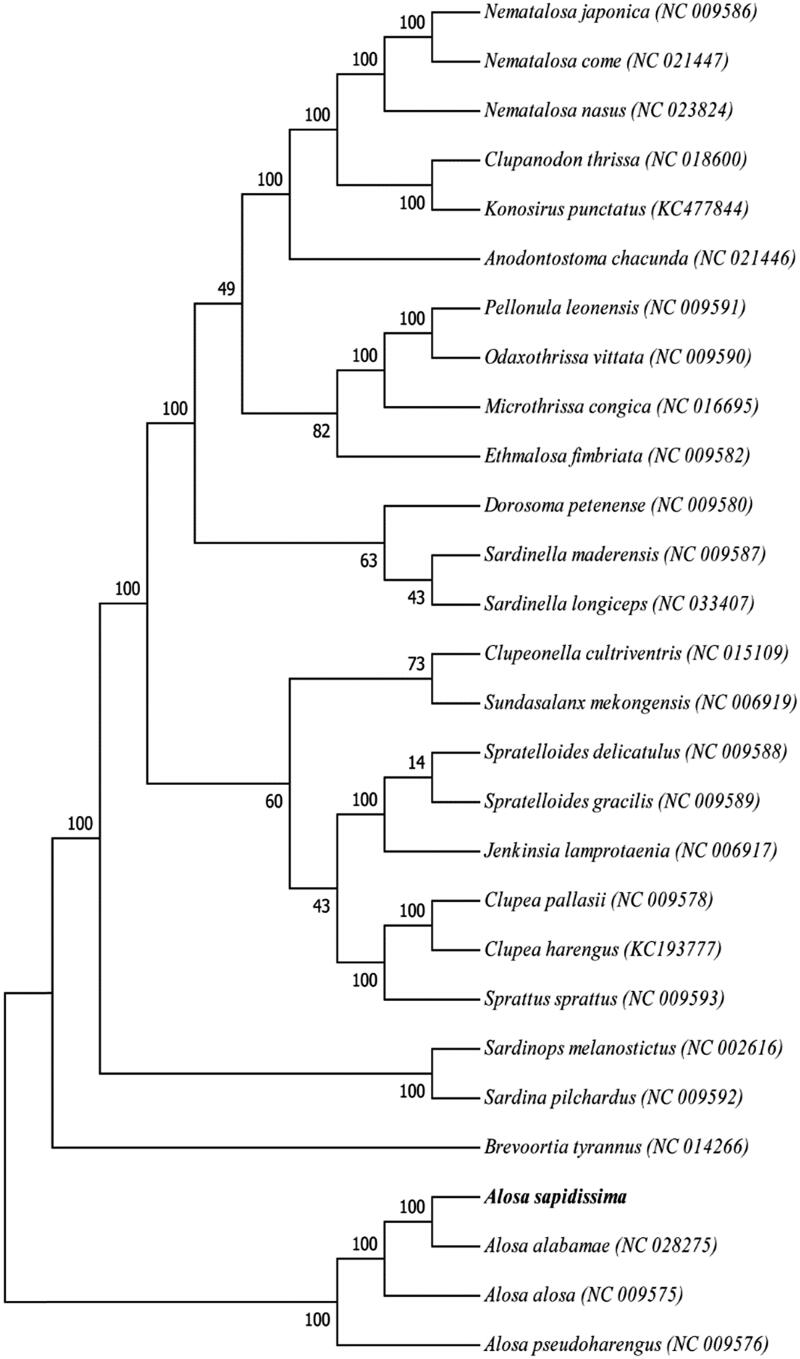
The ML phylogenetic tree of Perciformes species. Numbers on each node are bootstrap values of 100 replicates.
